# Sex related differences in the characteristics and outcomes of heart failure: A sub analysis of heart failure disease management study

**DOI:** 10.3389/fcvm.2022.1012361

**Published:** 2022-11-01

**Authors:** Avishay Grupper, Dov Freimark, Havi Murad, Liraz Olmer, Michal Benderly, Arnona Ziv, Nurit Friedman, Galit Kaufman, Haim Silber, Ofra Kalter-Leibovici

**Affiliations:** ^1^Leviev Heart Center, Ramat Gan, Israel; ^2^Sheba Medical Center, Ramat Gan, Israel; ^3^Sackler Faculty of Medicine, Tel Aviv University, Tel Aviv, Israel; ^4^The Gertner Institute for Epidemiology and Health Policy Research, Ramat Gan, Israel; ^5^Maccabi Health Services, Tel Aviv, Israel

**Keywords:** females, heart failure, sex differences, characteristics, outcome

## Abstract

**Aims:**

This is a sub-analysis of a randomized controlled trial on heart failure (HF) disease management (DM) in which patients with HF (*N* = 1,360; 27.5% women) were assigned randomly to DM (*N* = 682) or usual care (UC) (*N* = 678). Study intervention did not significantly affect the rate of hospital admissions or mortality. This study evaluates sex-related differences in baseline characteristics, clinical manifestations, adherence to treatment and outcomes among the study cohort.

**Methods:**

Association between sex and hospital admissions and mortality was tested in multivariable models adjusted for the patients’ baseline characteristics. The primary composite outcome of the study included time to first HF hospitalization or all-cause mortality. Secondary composite outcome included number of hospital admissions and days of hospitalization, for HF and all other causes.

**Results:**

Compared to males, females recruited in the study were on average 3 years older [median age 72 (62, 78) vs. 75 (65, 82), *p* = 0.001], with higher prevalence of preserved left ventricular function (LVEF ≥50%) and lower frequency of ischemic heart disease (IHD) (*p* ≤ 0.001). Females had shorter 6-min walking distance and worse quality of life and depression scores at baseline (*p* < 0.001). The proportion of patients receiving HF recommended medical treatment was similar among females and males. During a median follow-up of 2.7 years (range: 0–5), there were no significant differences between females and males with respect to the time elapsed until the study primary endpoint and its components in univariate analysis [557 (56.5%) males and 218 (58.3%) females were hospitalized for HF or died for any cause; *p* > 0.05]. Multivariable analysis showed that females were significantly less likely than males to experience the primary outcome [adjusted hazard ratio (HR) = 0.835, 95% CI: 0.699, 0.998] or to die from any cause [adjusted HR = 0.712; 95%CI: 0.560, 0.901]. The sex-related mortality differences were especially significant among patients with non-preserved EF, with IHD or with recent HF hospitalization. Females also had lower rates of all-cause hospital admissions [adjusted rate ratio = 0.798; 95%CI: 0.705, 0.904] and were more likely to adhere to HF medical therapy compared to males.

**Conclusion:**

Females with HF fare better than men. Sex related differences were not explained by baseline and morbidity-related characteristics or adherence to medical treatment.

## Introduction

Heart failure (HF) incidence is increasing in both males and females and becoming a major and growing problem globally. There are significant sex related differences in HF etiology, pathophysiologic mechanisms, clinical expression and age at disease onset, as females are more likely to be diagnosed with HF at an older age compared to males (1–5). One of the most notable sex-related differences in HF is that females have higher incidence of HF with preserved ejection fraction (HFpEF) which presents a greater diagnosis challenge with limited data to guide treatment options.

Age, sex and comorbidities are important considerations in HF outcomes and may be closely related to cardiovascular as well as non-cardiovascular events, leading to hospitalizations and mortality. There are conflicting data regarding sex-related differences in mortality among HF patients. While some studies reported a better survival for females compared to males (5–8), other studies showed no difference in mortality after adjusting for age and disease etiology (9–11). Previous epidemiological studies have been limited by retrospective design, underrepresentation of females, or exclusion of several comorbidities or HFpEF patients (12).

Our study aims were to examine sex-related differences in baseline characteristics, clinical manifestations, adherence to treatment and outcomes in a large cohort of HF patients who participated in a prospective randomized controlled trial on HF disease management (DM).

## Materials and methods

### Study design

This is a sub-analysis of a multicenter, open-labeled, prospective, randomized controlled trial on the effect of a DM program in community-dwelling patients with HF. The study design and methods were described elsewhere (13). In brief, the study population included adult patients (age ≥18 years), with symptomatic chronic HF [New York Heart Association (NYHA) functional class II to IV], recruited either from the community (62%) or after recent hospitalization for HF exacerbation (HHF) (38%). The study cohort was recruited between 2007 and 2012 and followed until death or end of study (2015). Patients with severe comorbidity or disability, e.g., Alzheimer’s disease, respiratory insufficiency, severe liver disease, metastatic cancer, HIV/AIDS or paraplegia/hemiplegia, were excluded. All participants were insured by Maccabi Health Services, the second largest health plan in Israel. HF diagnosis was based on typical signs and symptoms and objective echocardiographic evidence of functional or structural cardiac abnormality at rest. Patients were eligible regardless of their left ventricular ejection fraction (LVEF). Patients were randomized either to nurse-led DM intervention, or usual care (UC). The Sheba Medical Center and Maccabi Health Services research ethics committees approved the study protocol, and all patients signed a written informed consent form before randomization.

Baseline assessment included a physician-filled questionnaire on patient’s clinical characteristics, 6-min walk-test, NYHA classification, health-related quality of life (HRQoL) assessed with the 36-item short-form questionnaire (SF-36) (14) and depression symptoms assessed with the 9-item patient health depression scale (PHQ-9) (15). Information on purchase of medications for the management of HF [e.g., angiotensin converting enzyme inhibitors (ACEi), angiotensin receptor blockers (ARB), beta adrenergic receptor blocking agents (BB), mineralocorticoids receptor antagonists (MRA) and loop diuretics], chronic morbidity, laboratory test results, hospital admissions and deaths during follow-up were collected from the Maccabi Health Services database.

Hospital discharge summaries, available for 5,748 (99.7%) of 5,766 hospital admissions, were classified by two independent investigators after masking the patients’ identifying information. Each hospital admission was classified as either HF exacerbation related or as other cause for all non-HF related hospitalization. The primary composite outcome was the time elapsed until first HHF or all-cause mortality.

Overall, the study intervention (nurse-led DM intervention vs. UC) had a neutral effect with respect to the primary composite outcome, its individual components, the total number of hospital admissions and in-hospital days for HF and for any other cause (13).

The current study is a *post hoc* analysis of the original HF DM trial design to evaluate sex related differences in baseline characteristics, medical treatment, hospitalization, morbidity and mortality among the study cohort.

### Statistical analysis

Unadjusted comparisons of the baseline characteristics by sex were made using the chi square test for discrete variables. We used the unpaired *t*-test for comparisons of continuous variables with normal distribution, and the Mann-Whitney test for continuous variables with other distribution.

Unadjusted comparisons by sex with respect to the primary composite endpoint (time to first HHF or all-cause mortality) and its two individual components were made using the log-rank statistic.

The Cox proportional hazards model was used to compare males and females with respect to the primary composite endpoint and its components, adjusted for baseline characteristics [intervention type (DM or UC), age, 6-min walk test, NYHA classification, LVEF, source of referral (community or after recent HHF), BMI, chronic morbidity], and adherence to drug therapy for HF during follow-up, as time-dependent covariates. The time elapsed from recruitment until death from any cause and its 95% confidence interval (CI) was estimated for males and females, separately, standardized to the most common patients’ baseline profile, using an inverse transformation of the predicted survival curve at 75%. Predefined interactions between sex and baseline variables, including study group, LVEF, diabetes, underlying cause of HF (ischemic/non-ischemic), source of referral (community/post hospital admission), and NYHA functional class were also tested. The proportional hazards assumption for sex group was tested in all these models.

To compare hospital admission data recorded every 6 months for males and females, non-linear mixed models were used with a random intercept for each subject, allowing for correlation between repeated measurements of the same subject. Due to the over-dispersion commonly seen in hospital admission data, a negative binomial model was used. Using the NLMIXED procedure (SAS version 9.4), this method was implemented for the following secondary outcomes: number of hospital admissions and days of hospitalization, for HF and all other causes. These models were adjusted for baseline characteristics, adherence to drug therapy during follow-up and time since randomization in intervals of 6 months, and effectively dealt with the varying length of follow-up among individuals due to censoring. The coefficient of the sex group variable represented the log rate ratio for 6 months.

We used information from the baseline patient-questionnaire completed by the cardiologist and data from Maccabi Health Services databases to create a chronic morbidity score. This score included baseline renal failure and history of cancer, chronic obstructive pulmonary disease, acute myocardial infarction, stroke or transient ischemic attack and peripheral vascular disease. This score was designed to weight in the different comorbidities and evaluate their effect on prognosis.

Using the Maccabi Health Services database on drug purchases, adherence to medical therapy was defined as the number of days in a 6-month period covered by ACEi/ARB and BB therapy according to the defined daily dose, classified into 5 categories (1st category representing no treatment and 2nd–5th categories representing defined daily dose quartiles for patients taking the medication). The treatment odds ratio (OR) of attaining a higher level of coverage by drug therapy was estimated using an ordered logistic regression model within the non-linear mixed-model framework (NLMIXED Procedure in SAS). The assumption of proportional odds for each cut-point of the adherence variables was tested.

All analyses were carried out according to the intention-to-treat principle, at a critical two-sided significance level of 0.05.

## Results

### Study population

The study cohort included 1,360 patients, of whom 374 were females (27.5%). Table 1 presents patients’ baseline characteristics by sex. Compared to males, females recruited in this study were, on average, 3 years older [median age 72 (62, 78) vs. 75 (65, 82), *p* = 0.001], and presented with worse functional capacity, lower HRQoL scores and more depression symptoms (Table 1). Females were more likely than males to have HFpEF (LVEF ≥ 50%) and less likely to have HF due to IHD (Table 1).

**TABLE 1:** Baseline patients’ characteristics by sex.

	Men (*N* = 986)	Women (*N* = 374)	*P*-value
Age, years, median (interquartile range)	72 (62, 78)	75 (65, 82)	0.001
NYHA class, *N* (%)			0.058*
I	7 (0.7)	2 (0.5)	
II	154 (15.7)	43 (11.5)	
III	773 (78.6)	298 (79.9)	
IV	50 (5.1)	30 (8.0)	
LVEF (%), median (interquartile range)	30 (25, 40)	40 (30, 60)	< 0.001
LVEF category, *N* (%):			< 0.001
Preserved (LVEF ≥50%)	148 (15.2)	136 (36.7)	
Mildly reduced (LVEF 40–49%)	134 (13.8)	47 (12.7)	
Reduced (LVEF < 40%)	690 (71.0)	173 (46.6)	
Ischemic heart disease, *N* (%)	776 (78.7)	190 (50.8)	< 0.001
6-min. walk test, m, median (interquartile range)	216 (100, 320)	118.5 (50, 200)	< 0.001
Health-related quality of life, median (interquartile range):			
Mental score	48.2 (38.8, 59.7)	43.8 (35.3, 53.4)	< 0.001
Physical score	42.3 (30.6, 57.1)	32.8 (23.7, 44.3)	< 0.001
Depression symptoms (PHQ9 score ≥10), N (%)	373 (38.3)	203 (55.0%)	< 0.001
BMI, kg/m^2^, median (interquartile range)	29.0 (25.7, 32.3)	30.3 (26.2, 35.2)	< 0.001
BMI category, *N* (%):			< 0.001
<18.5 kg/m^2^	5 (0.5)	5 (1.3)	
18.5–24.9 kg/m^2^	196 (19.9)	71 (19.0)	
25–29.9 kg/m^2^	372 (37.8)	102 (27.3)	
30–34.9 kg/m^2^	288 (29.2)	100 (26.7)	
≥35 kg/m^2^	124 (12.6)	96 (25.7)	
Co-morbidity:	5 (0.5)	5 (1.3)	
Diabetes, *N* (%)	508 (51.6)	185 (49.7)	0.543
Hypertension, *N* (%)	721 (73.3)	289 (77.7)	0.108
Dyslipidemia, *N* (%)	767 (77.9)	274 (73.7)	0.113
COPD, *N* (%)	178 (18.1)	49 (13.2)	0.034
Myocardial infarction, *N* (%)	701 (71.2)	158 (42.5)	< 0.001
Stroke, *N* (%)	152 (15.4)	57 (15.3)	1.0
Recent hospital admission for HF exacerbation, *N* (%)	362 (36.7)	159 (42.5)	0.053
Hemoglobin (gr/dL), mean (SD)	13.0 (11.8, 14.1)	12.0 (11.1, 12.9)	< 0.001
Renal failure**, *N* (%)	536 (55.2)	219 (60.5)	0.093
Drug treatment at baseline, *N* (%):			
ACE-I/ARB’s	834 (84.6)	303 (81.0)	0.113
Beta adrenergic receptor blockers	829 (84.1)	307 (82.1)	0.377
Aldosterone antagonists	383 (38.8)	136 (36.4)	0.401
Loop diuretics	871 (88.3)	337 (90.1)	0.355
Statins	799 (81.0)	262 (70.1)	< 0.001
Anti-aggregants	730 (74.0)	235 (62.8)	< 0.001

NYHA, New-York Heart Association; LVEF, left ventricular ejection fraction; BMI, body mass index; COPD, chronic obstructive pulmonary disease; HF, heart failure; ACE-I/ARBs, angiotensin converting enzyme inhibitors or angiotensin-receptor blockers.

*Fisher’s exact test, ** eGFR < 60 mL/min/1.73 m^2^.

At recruitment, there were no sex-related differences with respect to treatment with guidelines directed HF medications (ACEi/ARB, BB, MRA and loop diuretics), but females were less likely than males to be treated with statins and anti-aggregants (Table 1).

### Primary endpoint and its components

**FIGURE 1:**
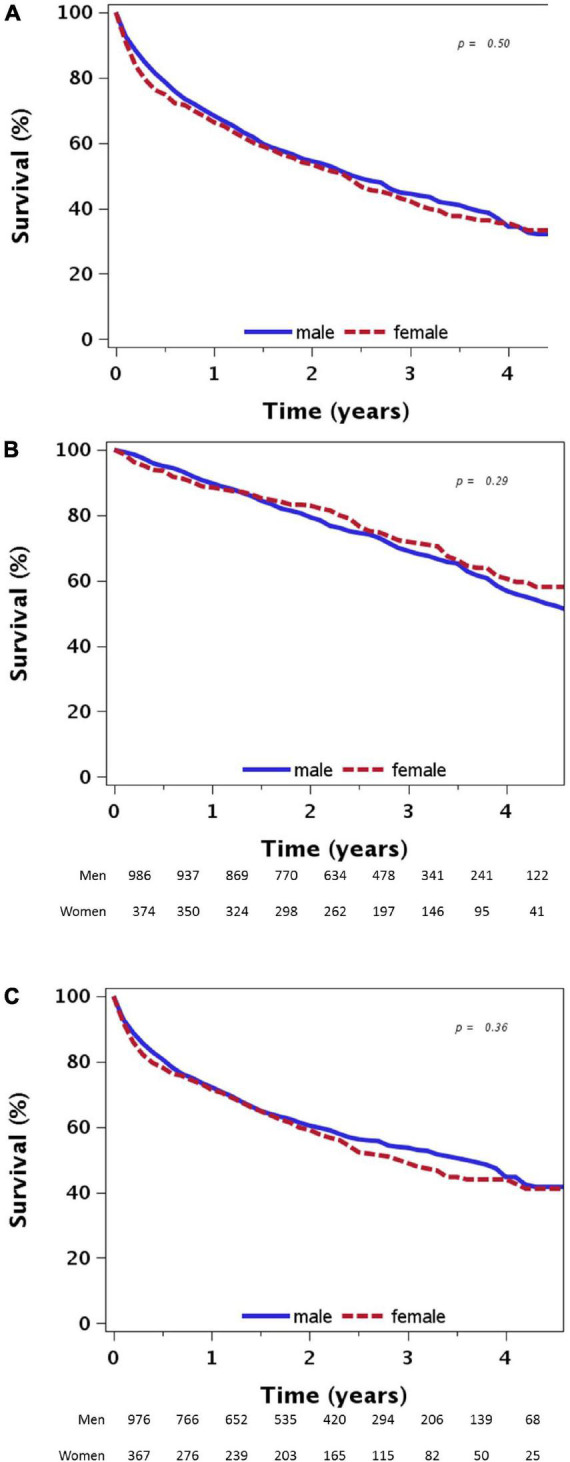
**(A)** Time to first heart failure hospitalization or death from any cause by sex. **(B)** Time to death from any cause by sex. **(C)** Time to first heart failure hospitalization by sex.

During a median follow-up of 2.7 years (range: 0–5), 557 (56.5%) males and 218 (58.3%) females had a primary composite endpoint event (HHF or all-cause mortality), and 334 (33.9%) males and 116 (31.0%) females died from any cause. The median (interquartile range) time elapsed until a primary endpoint event was 1.9 years (0.6, 2.9) among males, and 1.9 years (0.5, 3.0) among females (*p* = 0.5; Figure 1A). There were no significant differences between females and males with respect to the time elapsed until the study primary endpoint and its components in univariate analysis (*p* > 0.05 for all, Figures 1A–C).

Table 2 presents the effect of sex on the primary outcome and its components in several multivariable Cox proportional hazard models adjusted for several prognostic related baseline characteristics. The adjusted multivariable analysis demonstrated that females were less likely to experience a primary endpoint event during follow-up compared to male patients (HR = 0.842, 95% CI: 0.714, 0.993). The significant association between female sex and lower risk for the study primary outcomes remain even after adjustments for further clinical, laboratory and echocardiographic parameters as well as adherence to HF medical treatment (Table 2).

**TABLE 2:** Effect of sex on the primary outcome and its components (hazard ratio; 95% confidence interval)*.

Endpoint		Model-1	Model-2	Model-3
Number	Description			
1	First hospital admission for heart failure or death from any cause	0.842 (0.714, 0.993)	0.801 (0.669, 0.958)	0.835 (0.699, 0.998)
2	First hospital admission for heart failure	0.902 (0.749, 1.084)	–	–
3	Death from any cause	0.661 (0.528, 0.827)	0.651 (0.510, 0.831)	0.712 (0.560, 0.901)

*-Cox proportional hazard models, adjusted for: Model-1: study arm; study center, and baseline age, New York Heart Association functional classification and 6-min. walk-test. Model-2: model-1 variables plus source of referral (community or after recent hospital admission for heart failure), and baseline left ventricular ejection fraction, hemoglobin, body mass index and chronic morbidity score; study center was removed from the model since its association with the endpoint was not statistically significant. Model-3: model-2 variables plus adherence to medical treatment with angiotensin converting enzyme inhibitors/angiotensin receptor blockers, beta adrenergic blockers; aldosterone antagonists and loop diuretics (entered as time-dependent covariates).

Although there were no significant sex-based differences with respect to the time elapsed until death in univariate analysis (*p* = 0.29, Figure 1B), in multivariable analysis adjusted for a list of covariates, females were 30% less likely than males to die from any cause during follow-up (HR = 0.712, 95%CI: 0.560, 0.901; Table 2).

**FIGURE 2:**
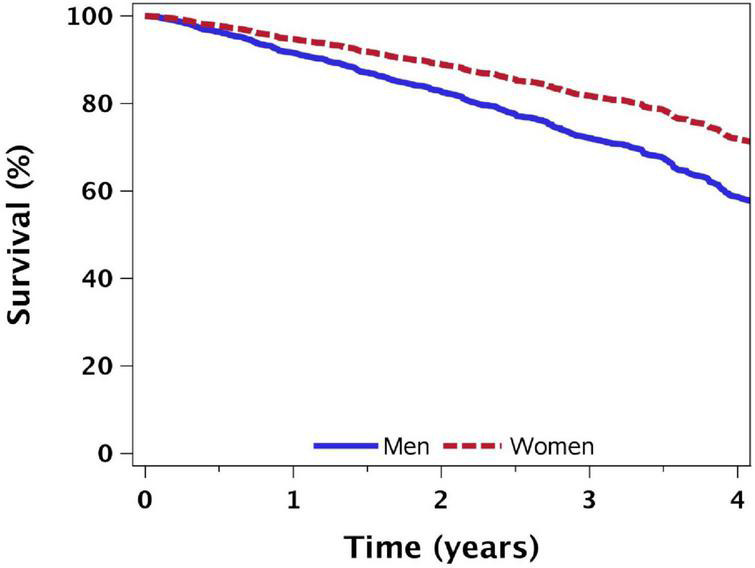
Sex differences in time-to death from all causes: results from Cox proportional hazards model*. *Adjusted for study arm (control); age = 72.5 years; NYHA functional classification III; 6-min walking distance: 180 m; referred from the community, with median comorbidity score and hemoglobin baseline values.

The time elapsed until 25% of the participants with a typical profile (age: 72.5 years, assigned to the control intervention, recruited from the community, HF due to IHD, NYHA functional class III, and median baseline values of comorbidity score, hemoglobin and 6-min walking distance at entry) died from any cause was 2.75 years (95%CI: 2.40, 3.23) among males and 3.80 years (95%CI: 3.29, 4.46) among female patients (Figure 2).

There was no significant difference between females and males with respect to the time elapsed until first HHF during follow-up, both in univariate analysis (*p* = 0.36, Figure 1C) and in multivariable analysis (Table 2).

**FIGURE 3:**
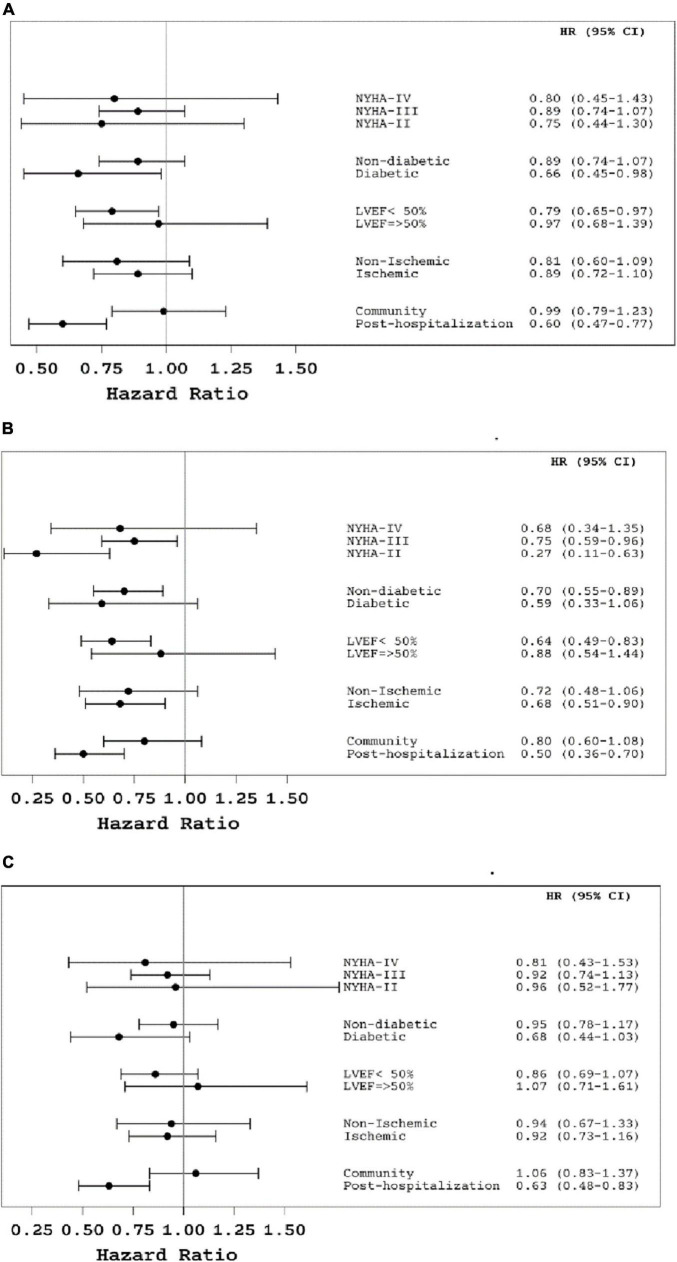
**(A)** The effect of sex on the primary composite outcome (time to first heart failure hospitalization or death from all causes) by sub-groups of patients*. **(B)** The effect of female sex on death from all causes by sub-groups of patients*. **(C)** The effect of female sex on the time until first heart failure hospitalization by sub-groups of patients*. *The information on the effect of sex in subgroups of patients was derived from Cox proportional hazard models, adjusted for age, study group, heart failure center, and baseline values of NYHA functional class and 6-min walk test.

None of the predefined interactions between sex and baseline variables with respect to the primary endpoint and its individual components were found statistically significant. Nevertheless, females with non-preserved ejection fraction (LVEF < 50%), after recent HHF, or with HF due to IHD were less likely to die from any cause during follow-up, than males with the same attributes (Figures 3A–C).

### Secondary endpoints

Table 3 presents the effect of sex on the total number of all caused hospital admissions during follow-up. In a multivariable analysis adjusted for study group and other baseline characteristics females had significant lower rates of hospital admissions and in-hospital days for all causes during follow-up compared to males [rate ratio (95%CI): 0.798; (0.705, 0.904), and 0.801 (0.661, 0.970), respectively]. Compared to a male patient, on average, a female patient had about 0.6 fewer hospital admissions for any cause during the first 2 years of follow-up (95%CI, −0.9 to −0.3; *p* = 0.0007), adjusted for age, study group, study period, year of recruitment, source of referral, baseline NYHA functional class and 6-min, walking distance, BMI and chronic morbidity score.

**TABLE 3:** Effect of sex on the number of hospital admissions for all causes during follow-up.

Variables adjusted for in the model	Adjusted rate ratio (95% confidence interval)	
	Number of hospital admissions for all causes	Number of in-hospital days for all causes
Model-1: Age, study group, study period, year at recruitment, source of referral*, baseline NYHA and 6-min walk test	0.762 (0.671, 0.865)	0.717 (0.581, 0.886)
Model-2: model-1 variables plus baseline left ventricular ejection fraction, body mass index, chronic morbidity index	0.787 (0.692, 0.895)	0.787 (0.644, 0.941)
Model-3: model-2 variables plus adherence to medical treatment during follow-up*.	0.798 (0.705, 0.904)	0.801 (0.661, 0.970)

*Adherence to treatment with angiotensin converting enzyme inhibitors or angiotensin receptor blockers; beta-adrenergic receptor blockers; aldosterone antagonists, and loop diuretics.

Female patients were more likely to adhere to medical therapy with ACEi or ARB and BB during follow up period compared to males [adjusted (OR) (95%CI): 1.461 (1.130, 1.891), and 1.374 (1.093, 1.726), respectively].

## Discussion

In this study, we investigated sex-related differences in demographic and clinical characteristics, treatment and long-term outcomes among a large cohort of HF patients recruited for a HF DM prospective randomized trial. Our findings confirmed sex-related differences in patients’ clinical profile and HF pathophysiology among a study population with a median age above 70 years old. The crude risk of morbidity and mortality represented by the study composite endpoint of HHF and all-cause mortality was similar between female and male patients. However, after adjustments for differences in baseline characteristics observed between the groups, females had a better prognosis than males, with a lower risk to experience a primary endpoint event during follow-up. This finding was attributed mainly to reduced risk of all-cause mortality among HF female patients. The sex-related mortality differences were especially significant among patients with non-preserved EF, with IHD or with recent HHF. We also found significant sex-related differences in the number of all-cause hospitalizations and in-hospital days, as female HF patients had lower overall admission rates and fewer in-hospital days compared to male patients. Our study demonstrates better adherence to HF medications during follow-up among females compared to males.

Our findings support data published in previous studies showing that there are significant differences in clinical phenotype of HF between females and males. The incidence of IHD and HF with reduced ejection fraction (HFrEF) is lower, and the prevalence of HFpEF is twice as high among females compared to males (1–5). Nevertheless, there was a similar incidence of HF with mildly reduced ejection fraction (HFmrEF) between both groups. Our study contributes to the existing data from previous studies demonstrating similar sex related differences in HF classification and etiology even in a cohort of HF patients with a median age above 70 years old.

Although many studies evaluated sex-related differences in mortality among HF patients, data are still conflicting. While several studies reported no difference in mortality (9–11) others demonstrated lower mortality among females compared to males HF patients. One possible explanation for these discrepancies is that higher incidence of HFpEF among female HF patients may be related to better long-term prognosis than males. This theory may be supported by a large meta-analysis of individual patient data, including almost 42,000 patients enrolled in 56 studies, showed that patients with HFpEF have 32% lower mortality rates than patients with HFrEF [adjusted HR: 0.68 (95%CI: 0.64, 0.71)] (16). However, several studies performed in HFpEF patients exclusively, showed that the association between female sex and lower mortality remained even when compared to HFpEF male patients (5, 17, 18). On the other hand, other studies demonstrated that the association between female sex and better prognosis was valid also in the group of patients with HFrEF. Dewan et al. compared sex-based outcomes among 12,058 HFrEF patients enrolled in 2 of the most recent and largest randomized controlled trials of pharmacological therapy in patients with HFrEF (19). Their analysis showed less frequent hospitalizations and longer survival among females with HFrEF compared to male patients. These results are consistent with other reports, demonstrating that the excess mortality risk associated with male sex was of similar magnitude across the LVEF spectrum (16, 20–22). Our results highlight the notion of better prognosis among female HF patients with significantly reduced morbidity and mortality even after adjustment for LVEF.

Another potential explanation for the sex related differences in outcomes is the higher incidence of IHD among male HF patients. In a meta-analysis from the Meta-Analysis Global Group in Chronic HF (MAGGIC) study group females with non-ischemic HF etiology had better prognosis than males with non-ischemic HF etiology, while among patients with HF due to IHD, the advantage of being a female was somewhat smaller (16). In contrast, IHD has not been independently associated with sex related outcome differences in HF patients (18, 20). We found that the lower all-cause mortality risk among females was consistent and even more significant among HF patients with IHD etiology.

Acute admission for HF exacerbation may represent deterioration in HF status and a worse prognosis (9, 10). Scrutinio et al. reported comparable mortality rates among females and males within 1-year after an acute admission for decompensated HF (11). The lack of sex-related mortality differences in this study and previous ones may be due to the sex-based differences related to the in-hospital treatment, as some of these studies reported a longer hospital stay for females, and lower rates of LVEF evaluation or implantable cardioverter-defibrillators implantation among female patients compared to males during HHF (9, 10). Our study sample, which included a large proportion of patients with stable HF recruited from outpatient clinics (62%), somewhat differs from previous studies. Nevertheless, our data rule out recent HHF as a confounder for sex related outcome differences since we have included this parameter in the adjusted multivariate analysis. In addition, our findings demonstrated significant lower all caused mortality in females compared to males among a subset of patients after a recent HHF.

Age and comorbidities may also be associated with poor outcome in HF patients. Previous HF studies have consistently demonstrated significant older age and higher incidence of multiple comorbidities in females compared to males (18, 20–22). Similarly, in our study females were on average 3 years older than males, which explains why the mortality risk between males and females differed only after age-adjustment. In addition, the lower rates of hospital admission from any cause among females in our cohort were observed even after adjustment for various clinical factors like: age, body mass index and other comorbidities, which may affect morbidity and were different between females and males. The survival benefit and lower hospital admission rates associated with female sex were also not explained by the better adherence to HF medical treatment among females compared to males in the multivariable analysis.

Although HF is a heterogeneous syndrome, varying by etiology and left ventricular function, HRQoL is impaired in many HF patients compared to patients without HF (23). However, there is considerable sex related variability in HRQoL despite similar disease severity. We found that females with HF have lower HRQoL scores and are more likely to describe depression symptoms than males. Previous studies also showed that females with HF have worse HRQoL scores compared to males, regardless of age or LVEF (19, 21, 24), partially due to lower social support or higher prevalence of depression (25–27). Similar sex-differences in HRQoL were reported also in other chronic diseases (28, 29). It appears that HF have a greater psychological impact on females than on males and improving HRQoL is an important goal in HF treatment. Thus, it is important to better identify and treat the clinical factors associated with worse HRQoL, and to provide social support along with medical treatment for HF patients, and especially among females.

### Study strength and limitations

One of the main strengths of our study is that all participants were insured by the same health plan provider, hence, all clinical and outcome data were available including documentation of medicines purchase.

Several limitations of this study should be acknowledged; First, our study population was enrolled in a clinical trial, so it is deemed selective due to inclusion and exclusion criteria. This may affect the external validity of the study since people with severe comorbidity were excluded. Second, the median age of our study cohort is above 70 years old, hence generalization of the results should be applied with caution to a younger patient population with HF. Third, the study was carried out in an environment with universal access to high-quality health care which may limit the generalizability of our findings in other health-care systems with barriers to universal access. Nevertheless, the extensive information on independent variables associated with HF outcomes that were controlled for in the multivariable models, the long-term follow-up and complete information of study endpoints provide significant contribution to our understanding of sex-related differences in HF outcomes.

## Conclusion

Females with HF have lower all-cause hospital admission and mortality rates. These sex-based differences were not explained by HF characteristics, comorbidity or adherence to HF recommended medical treatment. Our study provides novel information regarding better survival even among females with HFrEF and IHD compared to males. Despite better outcomes, females with HF reported more depression and lower HRQoL. The observed sex related differences in HF patients highlight the need for further research in this field and better representation of females in HF studies.

## Data availability statement

The original contributions presented in this study are included in the article/supplementary material, further inquiries can be directed to the corresponding author/s.

## Ethics statement

The studies involving human participants were reviewed and approved by the Sheba Medical Center Ethic Committee. The patients/participants provided their written informed consent to participate in this study.

## Author contributions

All authors has contributed significantly to the submitted work and seen and approved the final version of the manuscript.
